# Serum Neutrophil Gelatinase-Associated Lipocalin versus Serum Creatinine for the Prediction of Acute Kidney Injury after Liver Transplantation

**Published:** 2013-08-01

**Authors:** M. B. Khosravi, S. Milani, F. Kakaei

**Affiliations:** 1 *Anesthesiology and Critical Care Research Center*; 2 *Organ Transplant Research Center, Shiraz University of Medical Sciences, Shiraz, Iran*

**Keywords:** Acute kidney injury, Neutrophil gelatinase-associated lipocalin, Morbidity, Mortality, Liver transplantation

## Abstract

Background: Acute kidney injury (AKI) is a common complication after liver transplantation (LT), and considerably increases the morbidity and mortality of the procedure. The gold standard of measuring the kidney function, the serum creatinine level (sCr), has poor specificity and sensitivity for the early diagnosis of AKI. Novel biomarkers for the prediction or early diagnosis of AKI, would potentially increase the opportunities for therapeutic interventions.

Objective: To compare the diagnostic value of the standard renal marker, sCr and neutrophil gelatinase-associated lipocalin (NGAL) to predict AKI within 48 hours of LT.

Methods: During a 9-month period from 2010 to 2011, NGAL was measured two times in 90 patients who underwent LT from deceased donors—after induction of anesthesia (NGAL1) and 2 hours after reperfusion of the liver graft (NGAL2). Patients were grouped according to the presence of risk factors for developing AKI according to the Acute Kidney Injury Network criteria (increase of ≥0.3 mg/dL in plasma creatinine above the baseline value within 48 hours).

Results: 60 men and 30 women with mean±SD age of 40.2±14.2 years were included in this study. The incidence of AKI was 34% (95% CI: 24%–44%). The difference between the NGAL1 and NGAL2 (ΔNGAL) and the baseline NGAL concentration was predictive of AKI in all patients. Receiver operating characteristic (ROC*) *curve and area under curves (AUCs) of ΔNGAL and sCr levels during the first 48 hours of LT were similar in predicting AKI. The AUCs of the ΔNGAL to predict AKI was 0.64 (95% CI: 0.52–0.76). The development of AKI was significantly correlated with the number of units of fresh frozen plasma transfused intra-operatively (p=0.017) and cold ischemic time (p=0.042).

Conclusion: Serum NGAL concentrations obtained during surgery is a predictor of AKI in patients undergoing LT.

## INTRODUCTION

Orthotopic liver transplantation (OLT) is the definitive treatment for end-stage liver disease (ESLD). Acute kidney injury (AKI) is one of the most significant complications of OLT with high rates of morbidity and mortality. During the post-operative period, the rate of AKI varies between 12% and 70%; 71% of patients who developed AKI will require renal replacement therapy [1]. Although AKI are often multifactorial [2], the broad range in the incidence may be attributed to the lack of a uniform definition for AKI in this setting. Most recently, two similar definitions based on serum creatinine and urine output, *i.e.*, RIFLE (Risk-Injury-Failure-Loss-End-stage renal disease) and AKIN (Acute Kidney Injury Network), have been proposed and validated. According to the AKIN criteria, AKI is defined as either an absolute increase in plasma creatinine of at least 0.3 mg/dL (26.4 mmol/L) above the baseline value within 48 hours of OLT or a at least 50% increase in plasma creatinine during the prior seven days [3]. Recent findings suggest that even small increases in serum creatinine are predictive of non-renal outcomes; however, it is widely known that serum creatinine remains an insensitive and delayed marker of AKI [4]. Nevertheless, the treatment, in order to be effective, must be instituted very early after the initiating insult—well before the serum creatinine even begins to rise. Of several recently characterized novel renal biomarkers, neutrophil gelatinase-associated lipocalin (NGAL) appears to be the most promising [5, 6]. NGAL is a small protein expressed by neutrophils and various epithelial cells, including the renal proximal tubules. In the renal tubules, NGAL mRNA expression increases a few hours after renal injury [7]. Plasma NGAL is freely filtered in the renal glomeruli, but the vast majority of NGAL is reabsorbed via endocytosis in the proximal renal tubules. Thus, an increase in urinary NGAL excretion can occur only because of increased *de novo* synthesis of NGAL or proximal tubule cellular injury. NGAL is an acute phase reactant that may be released by neutrophils, macrophages, and other immune cells; thus, plasma concentrations of NGAL have been proposed as a biomarker for AKI [8]. Although the role of NGAL in non-transplant AKI is well recognized, its role in clinical transplantation is not still clear.

We conducted this study to re-evaluate the predictive value of serum NGAL for intra-operative AKI in patients undergoing OLT and investigated peri-operative variables that could have been associated with the development of AKI.

## MATERIALS AND METHODS

All adult patients who underwent OLT from deceased donors in Organ Transplant Center, Shiraz University of Medical Sciences, from September 2010 to January 2011, were included in this study. Patients who aged less than 18 years or had chronic hypertension, systemic infections, any systemic renal inflammatory conditions such as lupus erythematosis, anemia, hypoxia, malignancies, or pre-operative creatinine level >2 mg/dL, were excluded from the study. Also, those who were candidate for combined kidney-liver transplantation and those who received sirolimus or cyclosporine as their maintenance immunosuppressive regimen instead of tacrolimus were excluded from this study.

Blood samples for the determination of NGAL were drawn at two different times—immediately after the induction of anesthesia (*i.e.*, the baseline NGAL; NGAL1), and two hours after reperfusion (NGAL2). Plasma NGAL was measured using a commercially available ELISA kit (R&D systems, Minneapolis, MN, USA) according to the manufacturer’s instructions.

The anesthesia included the standard induction with thiopental (2–3 mg/kg), fentanyl (2–3 µg/kg), and a non-polarizing muscle relaxant or, in selected cases, succinylcholine. The anesthesia was maintained by isoflurane (1%–1.5%). Fentanyl was given either as a bolus or as continuous infusion as determined by the attending anesthesiologist. Patients were ventilated with a fraction of inspired oxygen of 0.5–1 L/min, tidal volume of 7–10 mL/kg, and a positive end-expiratory pressure of 0–5 cm H_2_O. Blood was transfused according to the patients hemodynamic or intraoperative bleeding, with a target hematocrit of 25%–28% and a platelet count >50,000/µL. Fresh frozen plasma (FFP) was administered according to serial thromboelastography results. All patients received 1000 mg intraoperative methylprednisolone for the induction of immunosuppressive regimen. All donor organ allografts were implanted either by standard caval replacement with bicaval anastomoses or by piggyback techniques. Veno-venous bypass was used in none of the patients.

Serum creatinine (Cr) levels were determined preoperatively (baseline) and then daily up to two days after the surgery. Serum Cr levels were used as a surrogate for renal function. AKI was defined as an absolute increase in plasma creatinine at least 0.3 mg/dL (26.4 mmol/L) above the baseline value within 48 hours of OLT.

Data analysis 

All data were analyzed by SPSS^®^ ver 16.0 for Windows^®^ (SPSS Inc. Chicago, IL, USA). All continuous data were expressed as mean±SD (range). For univariate analysis of risk factors for AKI, differences between the two groups were analyzed with Mann-Whitney U test for continuous variables and with χ^2 ^test for categorical variables. Receiver operating characteristic (ROC) curve was used to determine the cutoff point for plasma NGAL level to predict AKI. Based on the cutoff value, we then categorized patients into two groups of NGAL^–^ (NGAL<the cutoff value) and NGAL^+^ (NGAL≥the cutoff value). The patients were also stratified by their serum Cr according to the consensus diagnostic increases in serum creatinine defining AKI into sCREA^–^ and sCREA^+^. All tests were two-sided. P≤0.05 was considered statistically significant.

## RESULTS

A total of 90 (60 male and 30 female) patients were enrolled in this study. The mean±SD age of recipients was 40.2±14.3 years. They had a mean±SD body weight of 68.3±13.3 kg, and a MELD score of 22.0±6.2. Demographics of patients are shown in Table 1. Eight-one patients were operated by IVC sparing piggyback technique and nine by standard bicaval anastomosis method. The incidence of AKI (according to RIFLE criteria) was 34.4% (95% CI: 24%–44%).

**Table 1:** Demographic and causes of cirrhosis among the patients

Demographic characteristics	Mean±SD* (Median)
Age	40.2±14.2 (40.1)
Gender (male/female)	60 (67%)/30 (33%)
Weight	68.33±13.28 (65)
MELD score	22.0±6.2 (21)
Cause of Cirrhosis:	
HBV cirrhosis HCV cirrhosis Autoimmune Cryptogen PSC** Alcoholic Wilson Bud-Chiari Others	26 (29%) 2 (2%) 18 (20%) 19 (21%) 12 (13%) 1 (1%) 4 (4%) 5 (6%) 3 (3%)

* Data are presented as the mean±SD or number (%), MELD: Model for End-Stage Liver Disease

** Primary sclerosing cholangitis

Tables 2 and 3 depict peri-operative serum Cr levels, respective NGAL concentrations and peri-operative variables of all recipients. All patients with the baseline Cr level were included in the analysis.

**Table 2:** Univariate analysis of all baseline creatinine values

	**No** n=59(66%)	**Yes** n=31(34%)		p Value
Creatinine (mg/dL):				
Baseline	1.0±0.4	0.8±0.4		0.031
Day 1	0.8±0.4	1.4±0.9		0.027
Day 2	0.9±0.4	1.7±1.0		0.002
NGAL (ng/mL):				
1	93.0±63.6	99.5±52.9		0.524
2	193.0±132.7	238.4±153.7		0.508
Δ	109.0±95.0	83.0±74.0		0.029
Crystalloid (mL)	3564±991	3752±1277		0.951
Albumin(g)	63.1±22.2	73.3±26.8		0.074
Packed red cell (U)	2.0±1.9	3.4 ±3.3		0.083
Fresh frozen plasma (U)	4.0±3.0	5.4±4.7		0.017
Platelets (U)	1.5±0.5	2.7±0.9		0.697
Urine output (mL)	782±376	745±409		0.557
Pressors (µg)	140.0±82.3		78.2±52.4	0.551
Warm ischemic time (min)	41.2±7.3		43.4±9.8	0.255
Cold ischemic time (min)	449.0±172.8		524.0±112.5	0.042
Total bleeding (mL)	1641±1247		2232±1876	0.103
Ascitis (mL)	3797±2086		4014±2874	0.286
Duration of surgery (min)	348±57		373±59	0.078

s NGAL, Serum Neutrophil gelatinase-associated lipocalin; NOTE: Pressors include epinephrine or norepinephrine

The number of units of FFP transfused intra-operatively (p=0.017) and cold ischemic time (CIT) (p=0.042) were risk factors for AKI in OLT recipients (Table 2). There was no difference in MELD score between those who developed AKI and those who did not. Moreover, there was no difference in pre-operative aspartate aminotransferase (AST), alanine aminotransferase (ALT), alkaline phosphatase (Alp), albumin or bilirubin level between patients with and without AKI.

**Table 3:** Univariate analysis of all baseline Cr values

Preoperative Factors	No n=59(65.55)		Yes n= 31(34.45)		Pvalue
Serum Sodium (mmol/L)	137.05±6.26 (59)		135.83 ±7.10 (31)		0.507
Serum potassium (mmol/L)	4.37±0.62 (59)		4.40±0.57 (31)		0.619
Total bilirubin(μmol/L)	10.49±6.96 (59)		8.78±7.79 (31)		0.120
INR	2.12±1.75 (59)		2.24±1.42 (31)		0.912
MELD score		21.76±6.32 (59)	22.90±5.96(31)	0.251	
Hemoglobin (gm/dl)		11.27±2.07 (59)	11.62±1.83(31)	0.246	
Platelets count		117689±10000(58)	100451±10350(31)	0.355	

INR,International Normalized Ratio *؛* MELD score, The Model of End stage Liver Disease.

P value> 0.05 was not considered statistically significant.

The area under the curve (AUC) of the ROC curve of the difference NGAL1 from NGAL2 (ΔNGAL) to predict AKI was 0.64 (95% CI: 0.52–0.76); the best cutoff value (the point closest to sensitivity = specificity = 1) was 0.57 with a sensitivity of 71% and a specificity of 59% (Fig 1). The AUC for ROC curve of serum NGAL2 to predict AKI was 0.54 (Fig 2). ΔNGAL was predictive for AKI; serum NGAL2 concentration was not.

In our study, 17 (19%) patients were sCREA^–^/NGAL^–^, 42 (47%) were sCRE^–^/NGAL^+^, 13 (14%) were sCREA^+^/NGAL^–^ and 18 (20%) were sCREA^+^/NGAL^+^. Although, the incidence of AKI was 34.4%, six patients ultimately needed renal replacement therapy—four were sCREA^+^/NGAL^+^, and two were sCREA^+^/NGAL^–^.

NGAL1 was more than normal in nine (10%) of patients—in four NGAL2 was less than NGAL1 (Table 4).

**Table 4:** The specific characteristic of some the patients with NGAL2 < NGAL1

Case number	Sex	Weight (kg)	Age (yr)	Pre-operative diagnosis	MELD score	NGAL1*	NGAL2*
1	F	65	59	Autoimmune	26	234	224
2	M	78	27	Autoimmune	29	260	250
3	M	69	33	PSC†	23	244	236
4	M	90	46	HBV-cirrhosis	22	280	278
5	F	63	30	PSC	15	184	190
6	M	85	50	HBV-cirrhosis	20	186	202
7	F	77	18	PSC	40	238	224
8	F	62	22	Autoimmune	21	334	598
9	M	72	36	HBV-cirrhosis	23	190	152

* NGAL normal rang in healthy volunteers: 42-177 (ng/mL).

† PSC: Primary sclerosing cholangitis.

## DISCUSSION

The need for a simple, accurate, and minimally invasive marker for early diagnosis of AKI has been a limiting factor in clinical nephrology research and practice. The reference standard test for the diagnosis of AKI in current clinical practice is serum Cr level. Furthermore, prognostic scoring systems such as MELD, have shown that serum Cr is an important prognostic biomarker in cirrhotic patients, but its sensitivity and specificity is poor because it overestimates the renal function in patients with low muscle mass. The decreased hepatic production of Cr results in lower serum Cr levels [9, 10]. Recently, NGAL has been introduced as a sensitive and a marker for early diagnosis of AKI [11].

Parikh demonstrated that NGAL, an early biomarker of AKI after cardio-pulmonary bypass surgery, is markedly induced within 2–6 hour after the initiation of the operation in patients destined for AKI but it declines after 12 hour, when IL-18 and KIM-1 are easily detectable [12].

Previous studies have shown that NGAL levels reach the peak approximately 2 hours post-AKI [13]. Nonetheless, recent data show that NGAL is a very powerful predictor of AKI, even up to 12 hours after the transplantation [14].

In different clinical settings of AKI, Haase, *et al*, demonstrated that the cutoff value of NGAL concentration for prediction of AKI across all settings ranged from 100 to 270 ng/mL [15]. Recently, a study reported that the predictive value of NGAL increases with grade of AKI [16]. In this prospective study of critically ill patients, both transient (reversible) and prolonged (perhaps irreversible) increases in plasma creatinine were associated with increased urinary biomarker concentrations and these increased with duration of AKI [17].

While different definitions of AKI [18], various settings of AKI [19] and varying timings of NGAL measurement with regard to a renal insult have been used to assess the predictive value of NGAL level, a clear cutoff value for NGAL concentration for the early diagnosis of AKI has not yet been reported [20].

A recent analysis by Haase, *et al*, of cohort studies of patients with cardio-renal disease, demonstrated that an increase in plasma NGAL without an increase in plasma Cr can predict an increase in dialysis and mortality rate of the patients [21].

Nickolas, *et al*, confirmed that the intensive care unit patients with increased serum Cr at admission but negative NGAL (<130 ng/mL), rarely develop AKI and might not be planned for prolonged hospitalization [22]. This suggests that injury biomarkers have more important role in the classiﬁcation and management of AKI comparing with Cr.

Pre-renal AKI has recently been considered to represent a physiological response to under-perfusion. Its diagnosis is retrospective after a transient rise in plasma Cr, usually associated with evidence of altered tubular transport, particularly that of sodium. To test the reversibility of pre-renal AKI, Nejat, *et al*, measured urinary biomarkers of injury, *i.e.*, cystatin C, NGAL, c-glutamyl transpeptidase, IL-18, and kidney injury molecule 1 (KIM-1) at 0, 12, and 24 hours following ICU admission; it was shown that while the median concentrations of KIM-1, cystatin C, and IL-18 were significantly greater in those with pre-renal AKI compared to those without, NGAL and c-glutamyl transpeptidase concentrations were not significantly different. The results suggest that pre-renal AKI represents a milder form of injury. The reason why some biomarkers are increased requires further study [23]. Further studies are needed to correlate serum NGAL level with other established markers of renal injury—serum IL-6, IL-8, and urine NGAL—and renal function indices such as urine micro-albuminuria, and fractional excretion of sodium.

Our study has many limitations. First, the overall sample size was small and the incidence of post-operative AKI was significantly lower than that reported earlier, leaving a small pool of patients for analysis. This shortcoming diminished the statistical power of our study and precluded a more robust calculation of our biomarker’s predictive values. Second, serial NGAL levels may be more useful in the early diagnosis of AKI. Plasma NGAL measurements may be influenced by a number of coexisting variables including chronic kidney disease, chronic hypertension, systemic infections, inflammatory conditions, anemia, hypoxia and malignancies [24]. Patients with preoperative Cr level over 2 mg/dL, chronic hypertension, systemic infections, inflammatory conditions, anemia, hypoxia, malignancies, and combined liver-kidney transplantation, were excluded from our study. Recent studies demonstrated that higher plasma NGAL levels were associated with presence of anemia independent of estimated glomerular filtration rate, plasma high-sensitivity C-reactive protein, and myeloperoxidase levels. Therefore, plasma NGAL levels were inversely correlated with indices of anemia. Also, higher plasma NGAL levels were directly associated with indices of systemic inflammation and leukocyte activation [25]. Eirin, *et al*, found that chronic renovascular hypertension (RVH) is associated with elevated NGAL levels, likely due to ongoing kidney and systemic inflammation and ischemia [26].

Previous studies on AKI revealed that urine IL-18 and NGAL reach their peak almost six hours post-cardiac surgery [27]. Future studies will determine whether this biomarker would increase before serum Cr in patients undergoing OLT.

In conclusion, we found that ΔNGAL is a good marker for early detection of AKI after OLT. More studies on larger groups of patients, and combination of novel biomarkers of renal injury and function are needed to further elucidate the diagnostic values of these markers.

## Notes

**CONFILICT OF INTEREST:** None declared.

## Notes

**FINANCIAL SUPPORT:** Shiraz University of Medical Sciences
